# S05-3: Slovenian pathway to active and healthy ageing

**DOI:** 10.1093/eurpub/ckae114.220

**Published:** 2024-09-26

**Authors:** Tjasa Knific, Martina Horvat, Petra Nadrag, Pika Založnik

**Affiliations:** National Institute of Public Health; National Institute of Public Health; National Institute of Public Health; National Institute of Public Health

## Abstract

**Purpose:**

Slovenia's demographic indicators are rapidly increasing (21% older persons aged 65+ in 2023). Falls in Slovenia account for 53% of all deaths due to external causes and mortality of the elderly due to falls is 2.5 times higher in Slovenia than in other EU countries. Due to aforementioned reasons Slovenia modified the national prevention program Together for health for the needs of the elderly, focusing on the health sector, patients’ homes and local communities.

**Development:**

We modified the existing program, which generally promotes physical literacy for fall and frailty prevention. We successfully implemented national postgraduate training for health workers at the primary level of health care in the field of PA prescription and preventive treatment of the elderly (more than 700 specially trained health workers). We implemented multidisciplinary preventive treatment of those living at home, which is conducted by home nurses and is based on various geriatric syndromes, including falls.

**Implementation:**

National Institute of Public Health Slovenia is testing a new approach, which will bring new competencies for health, social workers and NGOs in falls screening and prescriptions of PA for falls prevention, which will bring treatment closer to the patient's home and will relieve the burden on Slovenian healthcare.

**Evaluation:**

In 2022, a total of 2117 patients were treated in PA for health workshops, 36% (n = 769) of them were over 65 years old. 58% were sufficiently physically active and results from national monitoring of aerobic capacity, strength, balance, and mobility show promising trends. Out of 13744 patients (mean age 81 years) who were home screened for falls in 2023, 49% were assessed as risky for falls.

**Conclusions:**

The program has shown improvement in the results in the majority of participating patients in all existing treatments for active and healthy aging, in the health sector and at the patients’ home. In coming years, we will focus on long term evaluation of PA behavior in the elderly who participate in the program. We expect closer cooperation with the sports sector in providing supportive activities in local communities when the PA treatment in the health care system has ended.

